# Conjugation with carbon nanotubes improves the performance of mesoporous silicon as Li-ion battery anode

**DOI:** 10.1038/s41598-020-62564-0

**Published:** 2020-03-27

**Authors:** Timo Ikonen, Nathiya Kalidas, Katja Lahtinen, Tommi Isoniemi, J. Jussi Toppari, Ester Vázquez, M. Antonia Herrero-Chamorro, José Luis G. Fierro, Tanja Kallio, Vesa-Pekka Lehto

**Affiliations:** 1 Department of Applied Physics, University of Eastern Finland, FI-70211 Kuopio, Finland; 2 Department of Chemistry, School of Chemical Technology, Aalto University, FI-00076 Aalto, Finland; 3 Department of Physics, Nanoscience Center, University of Jyväskylä, FI-40014 Jyväskylä, Finland; 4 Istituto Italiano di Tecnologia, 16163 Genova, Italy; 5 Departamento de Química Inorgánica, Orgánica y Bioquímica, Facultad de Ciencias y Tecnologías Químicas, Universidad de Castilla-La Mancha (UCLM) e Instituto Regional de Investigación Científica Aplicada (IRICA), 13071 Ciudad Real, Spain; 6 Institute of Catalysis and Petrochemistry, CSIC, Cantoblanco, 28049 Madrid, Spain

**Keywords:** Energy storage, Energy science and technology, Batteries, Carbon nanotubes and fullerenes, Electronic properties and materials

## Abstract

Carbon nanotubes can be utilized in several ways to enhance the performance of silicon-based anodes. In the present work, thermally carbonized mesoporous silicon (TCPSi) microparticles and single-walled carbon nanotubes (CNTs) are conjugated to create a hybrid material that performs as the Li-ion battery anode better than the physical mixture of TCPSi and CNTs. It is found out that the way the conjugation is done has an essential role in the performance of the anode. The conjugation should be made between negatively charged TCPSi and positively charged CNTs. Based on the electrochemical experiments it is concluded that the positive charges, i.e., excess amine groups of the hybrid material interfere with the diffusion of the lithium cations and thus they should be removed from the anode. Through the saturation of the excess positive amine groups on the CNTs with succinic anhydride, the performance of the hybrid material is even further enhanced.

## Introduction

Silicon is widely considered as the most promising anode material for Li-ion batteries because of its high theoretical capacity of 3579 mAh/g vs Li^+^ ^1–3^. The exploitation of the total capacity of silicon would mean high demand of lithium which cannot be met with the state-of-the-art cathodes. Lithium metal could be the solution if not the dendrite growth typically encountered^4,5^. The well-known drawbacks with silicon itself are the huge volumetric changes upon the lithiation and delithiation processes, and the subsequent loss of electrical contact during the delithiation phase as the material collapses^6^. New binders, binder architectures and electrolyte additives are being studied to mitigate this obstacle^7–9^. The current approaches to overcome the issue with silicon are typically based on different nanomaterials like nanoparticles, nanowires and thin films^10–12^. However, the complicated nanostructures circumvent the problem only on the laboratory scale due to expense of the material production and difficulties in the production scale-up^6^. Some studies have managed to successfully employ micrometer sized silicon with reasonable mass loadings^13,14^. Another viable option is to utilize mesoporous silicon (PSi) material that can accommodate most of the volumetric changes and make the anode more robust^15,16^.

Because of the semiconducting nature of silicon, the conductive additives to be added in the anode have an essential role in reaching good capacity with high charge/discharge rates (C rate) in the actual battery. Considering the high theoretical capacity of silicon, the current density that goes through the material is immense when compared with e.g. graphite especially at high C rate. In order to reduce the differences in the capacities obtained with different charging rates, carbon nanotubes (CNTs) can be added to link the particles together in the anode material even better^17^. The easiest way is to use a physical dispersion of silicon particles and CNTs^18^. In the present work it is found that there exists a specific way to conjugate these two materials together in order to get the optimal result. Using negatively charged silicon particles with positively charged CNTs is the most viable route to achieve high capacity even with high C rates. Moreover, it is discovered that the saturation of the excess positive charges in the hybrid material with succinic anhydride (SA) improves the performance even further.

## Materials and Methods

### Preparation and modification of PSi particles and CNTs

PSi microparticles were prepared in a similar fashion as reported previously^16^ and their pore size distribution was confirmed with gas adsorption (Micromeritics Tristar II 3020). The particles were sieved between 10 µm and 25 µm sieves (Precision Eforming) and their size was confirmed with laser diffraction (Malvern Mastersizer 2000 with Hydro 2000S accessory). Thermal carbonization (TC) of PSi particles was performed at elevated temperature under nitrogen atmosphere as described elsewhere^19^. Thermally carbonized porous silicon (TCPSi) particles were functionalized with either amine groups (-NH_2_)^20^ or carboxylic groups (-COOH)^21^. Amine groups were attached by first oxidizing the TCPSi surface (ammonia/hydrogen peroxide/water of the volume ratio 1/1/5 at 85 °C for 20 min, and subsequently after washing with water, hydrochloric acid/hydrogen peroxide/water 1/1/6 at 85 °C for 20 min, TCPSi-OX) and using (3-aminopropyl)triethoxysilane (APTES) afterwards (TCPSi-NH_2_)^21^. Carboxylic groups were added directly after the TC treatment by soaking the particles in undecylenic acid under nitrogen atmosphere and leaving them overnight at 120 °C (TCPSi-COOH)^21^. CNTs used in the present work were all single-walled carbon nanotubes (CNTs) with the length of 5–30 µm and the average diameter of 1.4 nm. CNTs and carboxylic functionalized CNTs (CNT-COOH) were used as received (Timesnano products TNS and TNSC, CNT purity >90%) while the amine modified CNTs (CNT-NH2) were prepared in a similar fashion as the amine modified TCPSi, i.e., through oxidation (CNT-OX) and APTES treatment.

The techniques utilized in preparation of the samples to combine TCPSi and CNTs (TCPSi-CNT) were as follows:

1° Physical mixing in ethanol.

2° Conjugation with carbodiimide compound (1-ethyl-3-(3-dimethylaminopropyl)carboimide (EDC) crosslinker and N-hydroxysuccimide (NHS), activator) where amine and carboxylic groups are bound together by forming an amide group^22^.

3° SA treatment was used to saturate the excess -NH_2_ groups into -COOH groups at the end of a hydrocarbon chains. First, TCPSi-CNT was dispersed to a buffer solution (0.9 g sodium bicarbonate, 0,1 g sodium carbonate, 50 ml water) to pH around 8. Succinic anhydride was added while mixing to get pH 7. Sodium hydroxide (0.1 M) was then added to increase pH to the range of 8–9. After 4 hours of mixing at room temperature, the sample was washed with water and ethanol.

In all the TCPSi-CNT samples, weight ratio of TCPSi to CNTs was 96:4. A typical batch size of the PSi sample was ca. 0.7 g. The samples are identified in Table 1.

**Table 1:** Different samples prepared by combing TCPSi and CNTs in various ways.

	−NH2	−COOH	Physical mixture (1°)	Conjugation (2°)	SA treatment (3°)
1. PM			x		
2. Func/PM	PSi	CNT	x		
3. Func/Conj	PSi	CNT		x	
4. Rev/Func/PM	CNT	PSi	x		
5. Rev/Func/Conj	CNT	PSi		x	
6. Func/Conj/SA	PSi	CNT		x	x
7. Rev/Func/Conj/SA	CNT	PSi		x	x

### Electrochemical measurements

Electrodes were prepared by mixing TCPSi or TCPSi-CNT hybrids with carboxymethyl cellulose (CMC, Sigma Aldrich), polyacrylic acid (PAA, Sigma Aldrich) and conductive carbon black (CB, C65, Timcal) in deionized water with 60/10/10/20 weight ratio, respectively^23^. CMC and PAA were used as binders and CB provided additional conductivity. The prepared slurry was spread on a copper foil using a coating machine (Doctorblade) set at wet thickness of 120 µm. Electrode sheet was dried at 150 °C for two hours in a vacuum oven. Electrodes were then cut and dried overnight in a vacuum oven at 110 °C. The mass loading of the active material in the electrodes was determined to be 1.2 mg/cm^2^. For the assembling of the cells, the electrodes were moved into argon glove box (Jacomex GP Campus). 2016 type coin cells (Hohsen) were assembled with a counter electrode of lithium metal (0.74 mm, Alfa Aesar), a fiberglass separator (GF/A, Whatman) and electrolyte (1 M LiPF_6_ in 1:1 ethylene carbonate (EC) dimethyl carbonate (DMC) (LP30, Merck)).

Cyclic voltammetry experiments were done with Autolab PGSTA302N and each sample was measured between 0.01 and 2 V with the scan rate of 0.1 mV/s for 6 cycles. Charge/discharge experiments were run with Neware battery testing device at room temperature with C-rates of 0.1 C, 0.2 C, 0.5 C and 1 C. Material was formatted with 0.03 C for one cycle and 4000 mA/g was used as a theoretical capacity. The mass of the TCPSi-CNT was considered in specific capacity calculations. In life cycle tests capacity was limited with time to 1200 mAh/g and a rate of 0.2 C was used after one formation cycle at 0.03 C. Voltage window of charge/discharge experiments was between 0.01 and 2 V. Electrochemical impedance spectroscopy (EIS) was performed with 5 mV amplitude over a frequency range of 10^5^–10^−2^ Hz with PGSTAT20 potentiostat (Metrohm Autolab) with FRA program using EL-CELL ECC-Combi three-electrode cell setup.

### Characterization

Scanning electron microscope (SEM) images were taken with Zeiss Sigma HD VP. X-ray photoelectron spectroscopy (XPS) studies were performed with Escalab VG 200 R equipped with a hemispherical electron spectrometer and MgKα radiation (hν = 1253.6 eV). The binding energies of photoelectrons were calibrated with respect to C 1 s peak at 284.8 eV. Malvern Zetasizer Nano ZS was used to determine the zeta potential of the chemically modified PSi nanoparticles. TAinstruments TA Q50 was used for thermogravimetric quantification of functional groups on PSi and CNTs with heating rate of 20 °C min^−1^ under nitrogen flow. FTIR measurements were done with Thermo Nicolet Nexus 8700 using potassium bromide tablets in transmission mode.

## Results and discussion

### Active materials characterization

**Figure 1:**
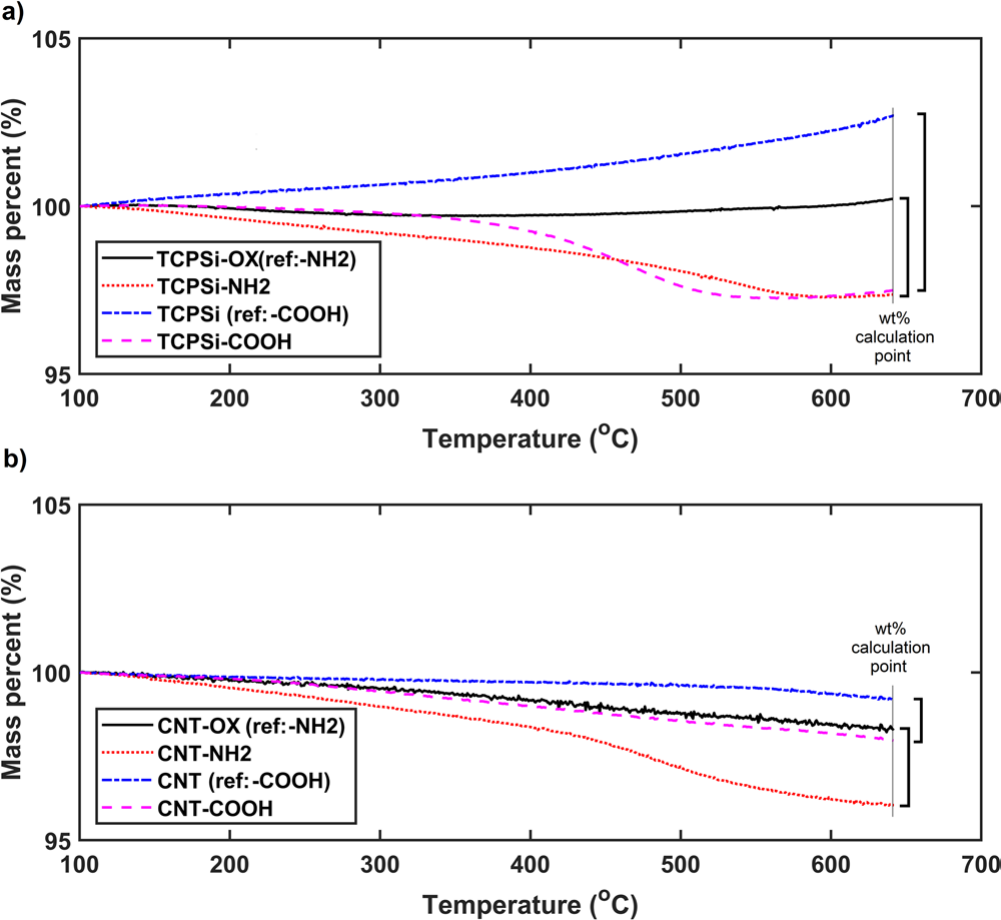
Thermogravimetric results of different TCPSi (**a**) and CNT (**b**) samples with reference samples. TCPSi-OX and CNT-OX have been oxidized (references for TCPSi-NH2 and CNT-NH_2_).

Pore size distributions (Fig. S1) and particle size distributions (Fig. S2) of TCPSi microparticles indicate pore size around 5 nm and mean particle diameter of 21 µm with specific surface areas between 155–165 m^2^/g. The amounts of functional groups on PSi and CNTs are characterized with thermogravimetry (TGA) (Fig. 1). Based on TGA, TCPSi-NH_2_ samples have 3 wt% and TCPSi-COOH 4 wt% of functional groups (Fig. 1a). Similarly, CNT-NH_2_ have 2 wt% and CNT-COOH 1 wt% of functional groups (Fig. 1b).

To confirm the successful conjugation between TCPSi and CNTs, XPS measurements are made to investigate the binding energies of the core electrons of the samples (Table 2). The deconvoluted XPS spectra are shown in Fig. S3.

**Table 2:** Core-electron binding energies of different TCPSi-CNT samples. The relative peak areas in percentage terms are given in parentheses.

Sample	C1s (eV)	N1s (eV)	O1s (eV)	Si2p (eV)
**Rev/Func/PM**	284.8 (78)	400.2 (44)	532.6	99.7(59)
	286.2 (22)	**401.7 (56)**		100.9 (28)
				102.7 (13)
**Rev/Func/Conj**	284.8 (68)	399.6 (39)	531.7	98.8 (57)
	286.0 (23)	**401.6 (61)**		100.2 (30)
	**288.2 (9)**			102.0 (13)
**Rev/Func/Conj/SA**	284.8 (67)	400.2	532.6	99.9 (53)
	286.1 (25)			101.0 (27)
	**288.1 (8)**			102.4 (20)

Specifically, the energies of C1s and N1s are of high interest. C1s peak at about 288 eV indicates amide (N-C=O) bond and is present in samples Rev/Func/Conj and Rev/Func/Conj/SA. The peak is absent from the non-conjugated (Rev/Func/PM) sample as expected. N1s peak at about 402 eV indicates a protonated nitrogen which is present in Rev/Func/Conj and Rev/Func/PM samples. This finding confirms that SA treatment is effective in saturating the excess amine groups. For further confirmation, FTIR indicated an amide bond in the form of C=O stretch at 1673 cm^−1^ for Func/Conj sample while the peak is absent for both CNT-COOH and Func/PM samples (Fig. S4). Furthermore, the atomic surface composition obtained with XPS (Table S1) reveals that SA treatment reduces the amount of nitrogen at the sample surface by a factor of four while increasing the content of oxygen and silicon. This is a strong indication of -COOH groups being added in the treatment at the cost of -NH_2_ groups. Conjugation, however, seems to increase the amount of surface nitrogen. This is partly due to formation of amide bonds but also because the used reagents (EDC, NHS) have secondary reactions during conjugation^24^.

**Figure 2:**
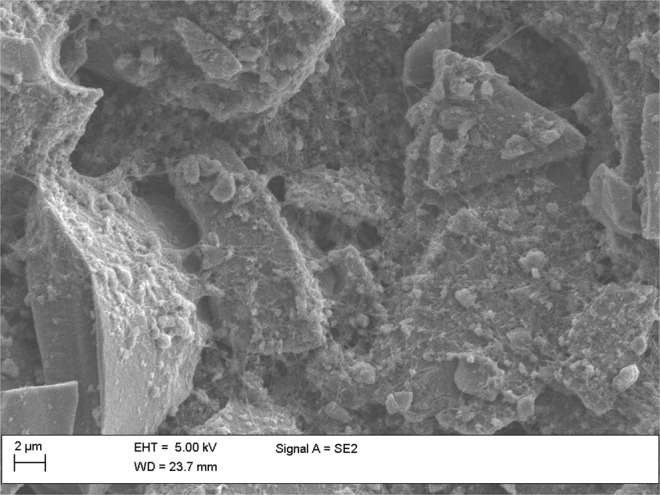
SEM image of Rev/Func/Conj/SA active material. TCPSi particles are bound together with CNTs at multiple points.

Three SEM images can be found in the Supplementary information depicting individual PSi particles (Fig. S5), CNTs on holey carbon grid (Fig. S6) and an image of individual PSi particle which is partially covered with CNTs (Fig. S7). The SEM image of the Rev/Func/Conj/SA active material is shown in Fig. 2. TCPSi-COOH particles are conjugated successfully with CNT-NH_2_ and subsequently treated with SA to saturate the excess -NH_2_ groups. Individual particles can be seen bound together by a web of CNTs from multiple points. This interlinked structure is beneficial for two reasons: First, even if the particles crack during the charge/discharge cycling, there is a high probability that CNTs even with a low amount of 4 wt% can keep the material connected to the current collector and to each other. In our preliminary experiments, 4 wt% of CNTs was found to be the optimal choice over 1 wt%, 10 wt% and 20 wt% of CNTs. The benefit is low amount of CNT agglomerates and good coverage over TCPSi. Second, CNTs have much higher conductivity being able to enhance the rate capability of the electrode.

To verify the effect of different surface modifications on the zeta potential of the TCPSi particles, separate nanoparticle batches of the materials are produced. The SA modified particles have the negative zeta potential of −70 mV whereas amine modified particles have the zeta potential of +31 mV (Fig. S8). The results, together with XPS atomic surface composition results, show that the amine groups can be effectively turned to carboxyl groups with the utilized SA treatment.

### Electrochemical results

**Figure 3:**
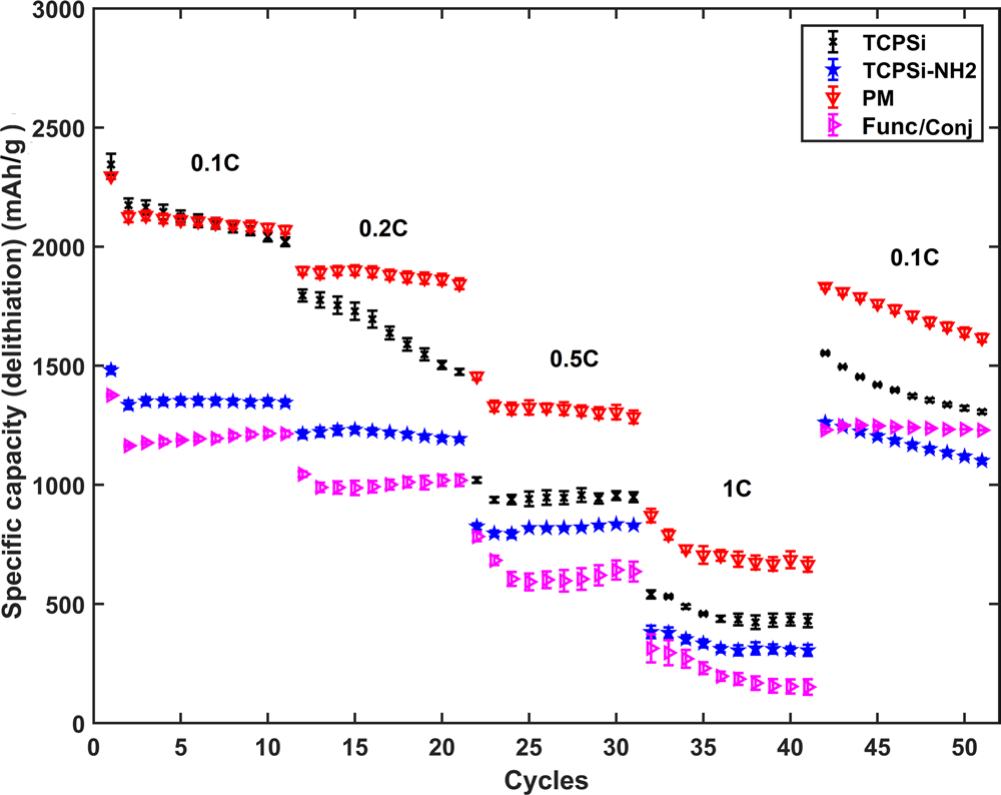
Galvanostatic rate capability results for the TCPSi, TCPSi-NH_2_, PM and Func/Conj samples. The results are shown with average value and standard error (n = 3).

To verify the role of CNTs in the performance of mesoporous silicon as the anode material, the rate capability properties of four different anodes are studied. CNTs are either physically mixed (PM) or chemically conjugated with TCPSi microparticles (Func/Conj). The references are the anodes made from microparticles of TCPSi and TCPSi-NH_2_ (Fig. 3). Contrary to our expectations, PM performs better than Func/Conj. Another interesting result is the poor performance of TCPSi-NH_2_ (Fig. 3). Positively charged -NH_2_ groups have a negative impact on the performance of the TCPSi material. This is contradictory to some earlier published results but in the present work the samples have much higher contents of functional groups than in the previously published papers (3 wt% vs 1 wt%^25^), which could explain the different result.

**Figure 4:**
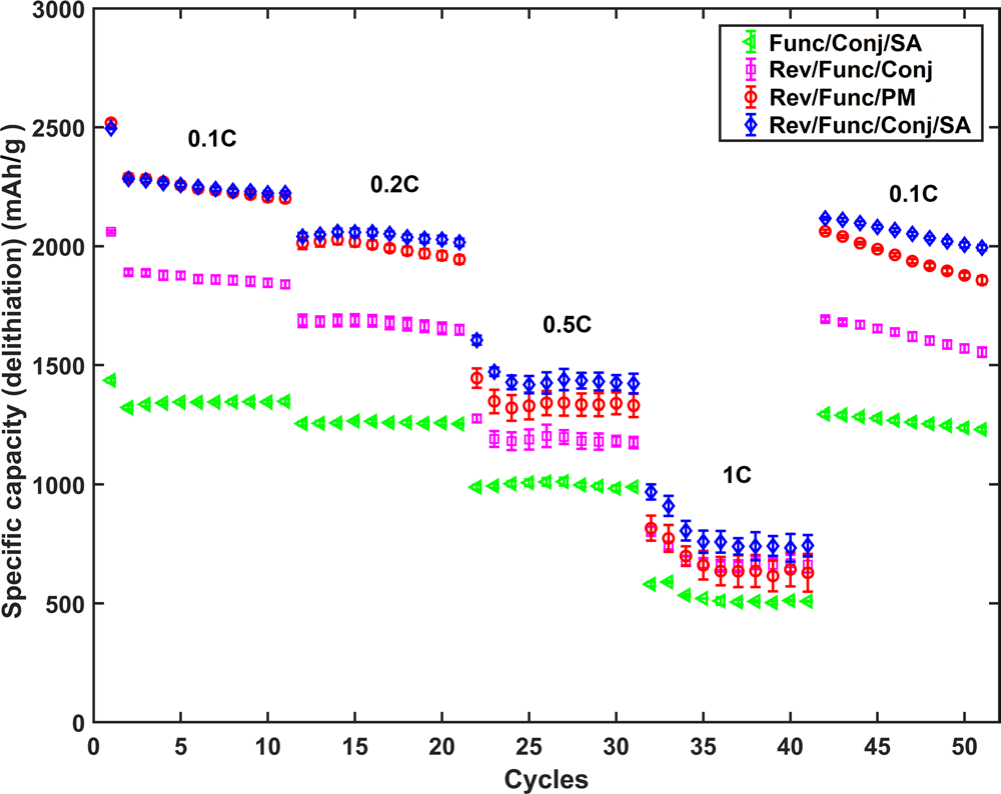
Galvanostatic rate capability results for the Func/Conj/SA, Rev/Func/Conj, Rev/Func/PM and Rev/Func/Conj/SA samples. The results are shown with average value and standard error (n = 3).

On this thought, the surface chemistries of TCPSi and CNTs are changed the other way around, i.e., TCPSi is modified with -COOH groups and CNTs with -NH_2_ groups (sample Rev/Func/Conj and Rev/Func/PM, Fig. 4). Furthermore, a known method from biochemistry is used to turn the excess amine groups into carboxylic groups by using SA after conjugation of TCPSi and CNTs (sample Func/Conj/SA and Rev/Func/Conj/SA, Fig. 4)^26^. To our knowledge, SA has not been used in the similar way previously but rather as an electrolyte additive^27,28^.

The SA treatment has a tremendous effect on the higher charge/discharge rates of 0.5 and 1 C when compared to the original functionalization. Furthermore, with SA treatment the sample outperforms also PM sample on all charge/discharge rates (Fig. S9). Rev/Func/Conj with reversed functional groups is a clear improvement over the original Func/Conj but not enough to surpass PM in specific capacity. However, Rev/Func/PM and Rev/Func/Conj/SA provide better performance than PM. The enhanced properties of Rev/Func/PM compared to Rev/Func/Conj are attributed to a more negative charge since conjugation of the sample will not only create amide bonds between the TCPSi-COOH surface and CNTs but also causes other nitrogen compounds to appear. After removing the excess amines of the hybrid material with SA, the best sample is created: Rev/Func/Conj/SA. This sample can provide 740 mAh/g even at 4 A/g charge/discharge rate with relatively high mass loading of 1.2 mg/cm^2^. Coulombic efficiency (CE) after formation is >99.3% for Rev/Func/Conj/SA and >99.1% for Rev/Func/Conj/PM.

**Figure 5:**
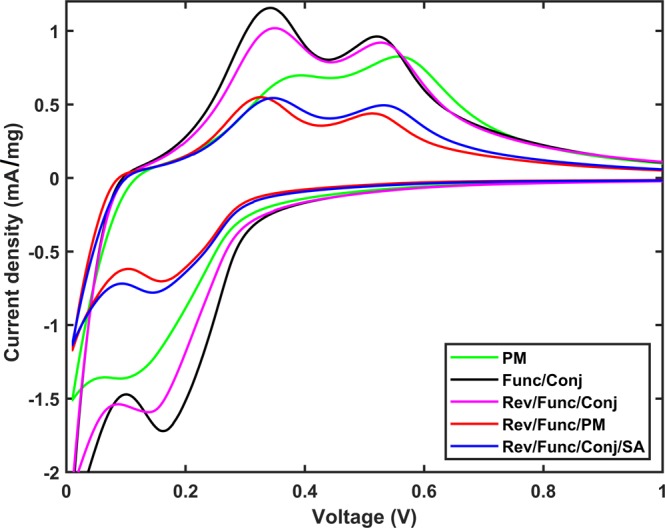
Cyclic voltammetry results for 6th cycle for samples PM, Func/Conj, Rev/Func/Conj, Rev/Func/PM and Rev/Func/Conj/SA.

The cyclic voltammetry (CV) spectra of the investigated materials are presented in Fig. 5. The capacity obtained from CV increased up to 6th cycle (Fig. S10). This is caused by the relatively large scan rate of the CV measurement (0.1 mV/s). As the cells are not formatted before the CV measurements, the electrode reaction does not occur completely during the first cycles. Therefore, to present stabilized spectra, the 6th cycles of the measurements are presented here for each sample.

Cyclic voltammetry measured for the five chosen samples reveals that in general the samples Rev/Func/PM and Rev/Func/Conj/SA have the best kinetics based on the half peak potentials, indicating that both lithiation and delithiation have been enhanced compared to physical mixing (Fig. 5, Table S2). For Rev/Func/Conj/SA, the delithiation kinetics is slightly worse than for Rev/Func/PM. Similarly, Func/Conj is worse than Rev/Func/Conj/SA even though Func/Conj has highest peak values regarding current density. Rev/Func/Conj and PM have overall the poorest kinetics when compared with the other three samples. The maximum difference in the half peak potentials within the five samples is around 50 mV which is quite large variation for individual peaks. This confirms once again the effect of surface functionalization.

**Figure 6:**
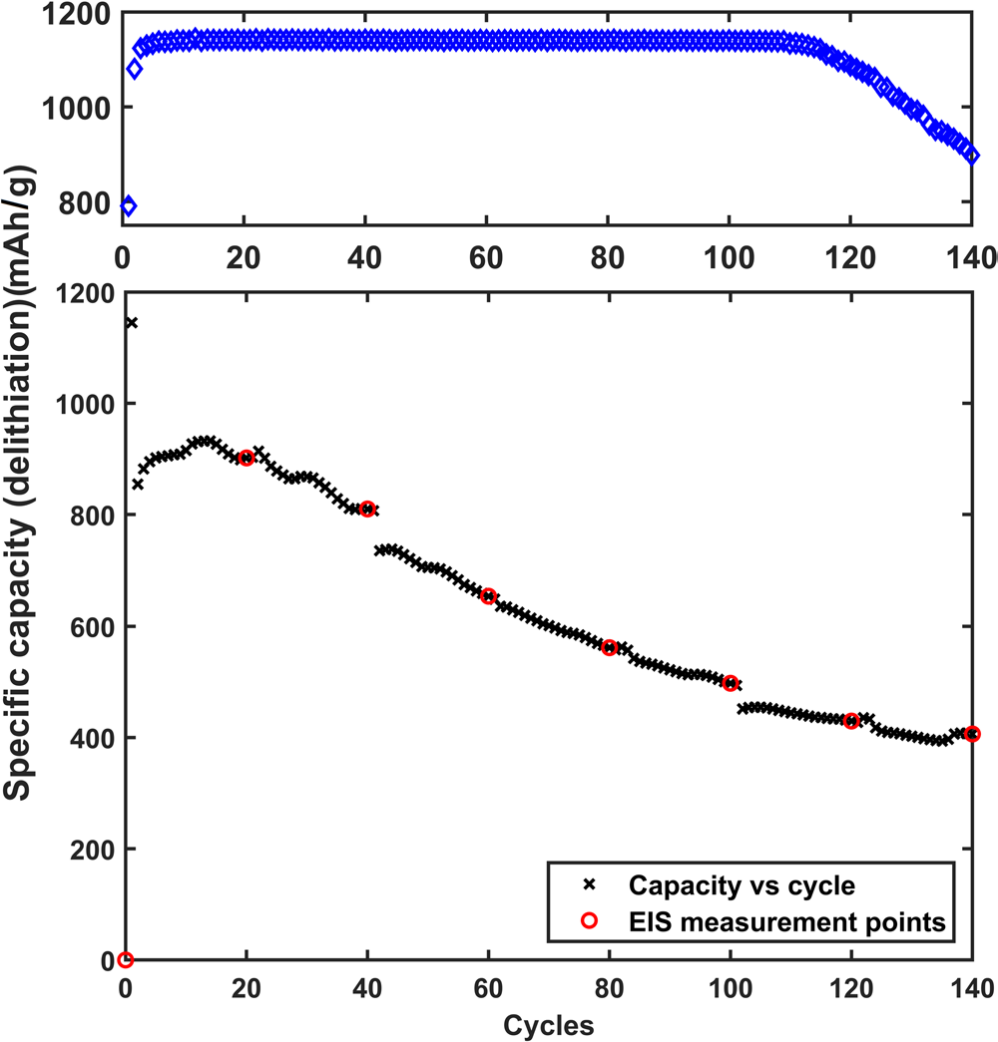
Top: Galvanostatic life cycling data of Rev/Func/Conj/SA. Bottom: Life cycling data of Rev/Func/Conj/SA for electrochemical impedance spectroscopy study. In both cases, capacity was limited and cycling rate was 0.2 C.

To further study the best performing sample, Rev/Func/Conj/SA, life cycle test and a separate life cycle test with EIS is performed (Fig. 6). The long-term cycling shows promising results with the stable capacity of approximately 1150 mAh/g up to 110 cycles (top part of Fig. 6) with CE over 99.4% after formation. As the long-term cycling was done in half-cells, it is quite likely that the sudden capacity decrease after 110 cycles is caused by the consumption of the organic solvent of the electrolyte. This phenomenon is accelerated by the SEI layer formation on the active material, and also on the metallic Li counter electrode. From cycle voltage profiles (Fig. S11) a typical shoulder corresponding to SEI formation is seen between 1000 mV and 250 mV on the formation cycle which is also visible in the CV results (Fig. S10).

The cycling data for impedance measurement is presented in the lower picture in Fig. 6. The capacity in this measurement is not stable and does not reach as high values as it does in the cycle life tests. There are two likely causes for this. First, the cell setup used in the EIS measurement cells is different with e.g. a thicker separator used to enable the use of a reference electrode in the 3-electrode setup. This could for example increase the resistance of the system, increasing the capacity decrease. A second cause is the disturbance caused by the EIS measurements. As the impedance was measured separately from the cycling, it had to be removed from the cycler every time for the EIS measurement. This could affect the cycling result.

**Figure 7:**
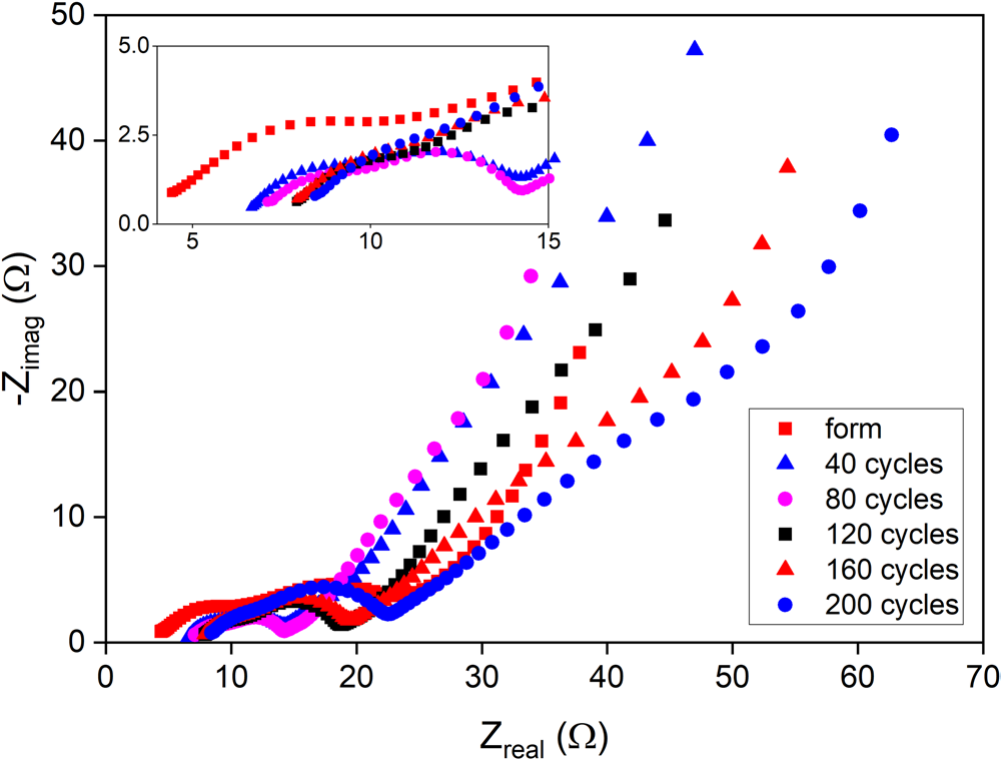
Nyquist plots for Rev/Func/Conj/SA sample on different cycles.

The Nyquist plots of the Rev/Func/Conj/SA sample at different cycle numbers are presented in Fig. 7. The chosen equivalent circuit (EC) for EIS is shown in Fig. S12 along with the experimental data and the fitted curve for the formatted Rev/Func/Conj/SA sample. The Nyquist plot (Fig. 7) consists of two semi-circles at high and middle frequencies and a tail at low frequencies.

Therefore, the EC consists of three parts in series where R_1_ depicts equivalent series resistance caused mostly by the electrolyte, R_2_ relates to the interfacial contact within the hybrid electrode, R_3_ relates to the charge transfer/double layer (CT/DL) resistance, and W_2_ represents the Li diffusion in the solid Si. Based on the equivalent circuit fit (Table 3) the electrolyte resistance/equivalent series resistance increases a lot during the first 40 cycles, the initial resistance being 4.2 ohms and the resistance at 40 cycles being 6.6 ohms. After this, however, the increase rate of the resistance slows down, and the equivalent series resistance after 200 cycles is 8.4 ohms. The increasing resistance over time is due to degradation of the organic solvent in the electrolyte.

**Table 3:** EC-fit based kinetic parameters for Rev/Func/Conj/SA sample.

Cycle (V)	0 (0.21)	40 (0.42)	80 (0.23)	120 (0.23)	160 (0.30)	200 (0.30)
Element						
**R**_**1**_ **(Ω)**	4.2	6.6	7.0	7.8	7.8	8.4
**R**_**2**_ **(Ω)**	7.8	4.0	3.9	4.6	4.8	5.1
**R**_**3**_ **(Ω)**	7.9	5.1	5.1	7.3	8.1	8.7

Interfacial contact R_2_ within hybrid electrodes is seen to be relatively large just after the formation. However, after that it decreases and starts to increase slowly upon cycling. The charge transfer/double layer (CT/DL) resistance R_3_ behaves similarly. The resistance drop after 40 cycles is obviously caused by the partial fracturing of the Si particles during the cycling. On the other hand, the smaller particles formed can give better contact with the current collector and result in decreased resistance especially when connected through CNTs. Smaller particles also offer shorter diffusion distances for Li, further lowering the resistance. Between 80 and 120 cycles both R2 and R3 increase significantly due to continuously degrading electrolyte.

## Conclusions

The TCPSi-CNT hybrid material produced through conjugation and the subsequent succinic anhydride treatment was found to be the best way to combine silicon and CNTs. Negatively charged silicon surface was beneficial for lithium-ion battery anodes giving the highest specific capacity. The observed phenomena related to the succinic anhydride treatment and its usage as an electrolyte additive needs to be further studied as SA seems to have benefits in at least two ways; actively as an electrolyte additive and passively as a reagent to change the surface chemistry as used in the present work. Regarding the function of high surface area battery materials, the surface chemistry and the polarity should be closely considered.

## Supplementary information

Supplementary information.
