# Supplementary material for: Conjugation with carbon nanotubes improves the performance of mesoporous silicon as Li-ion battery anode
Source: Sci Rep. 2020 Mar 27;10:5589. doi: 10.1038/s41598-020-62564-0 (PMC7101375; doi:10.1038/s41598-020-62564-0)
Supplement: Supplementary file 1 — Supplementary information. [file 41598_2020_62564_MOESM1_ESM.docx]

Supplementary information for

**Conjugation with carbon nanotubes improves the performance of mesoporous silicon as Li-ion battery anode**

Timo Ikonen^1^, Nathiya Kalidas^1^, Katja Lahtinen^2^, Tommi Isoniemi^3,4^, J. Jussi Toppari^3^, Ester Vázquez^5^, M. Antonia Herrero-Chamorro^5^, José Luis G. Fierro^6^, Tanja Kallio^2^, Vesa-Pekka Lehto^1,*^

^1^Department of Applied Physics, University of Eastern Finland, FI-70211 Kuopio, Finland

^2^Department of Chemistry, School of Chemical Technology, Aalto University, FI-00076 Aalto, Finland

^3^Department of Physics, Nanoscience Center, University of Jyväskylä, FI-40014 Jyväskylä, Finland

^4^Istituto Italiano di Tecnologia, 16163 Genova, Italy

^5^Departamento de Química Inorgánica, Orgánica y Bioquímica, Facultad de Ciencias y Tecnologías Químicas e Instituto Regional de Investigación Científica Aplicada (IRICA), 13071 Ciudad Real, Spain

^6^Institute of Catalysis and Petrochemistry, CSIC, Cantoblanco, 28049 Madrid, Spain

*Corresponding author: [vesa-pekka.lehto@uef.fi](mailto:vesa-pekka.lehto@uef.fi)


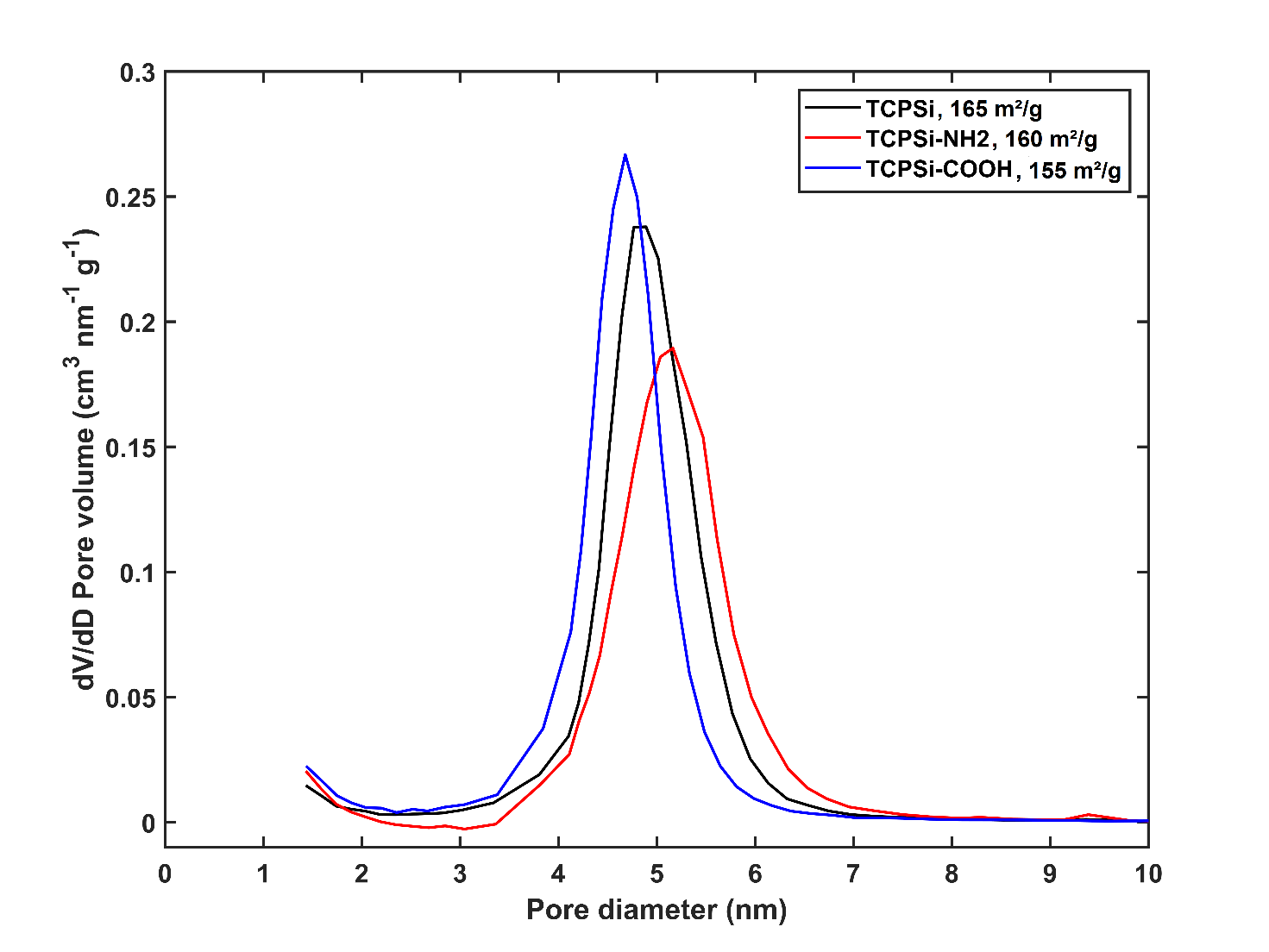


**Fig. S1.** Pore size distribution of TCPSi, TCPSi-NH2 and TCPSi-COOH particles. Legend includes BET specific surface area values (m^2^/g) for each sample type after sample name.


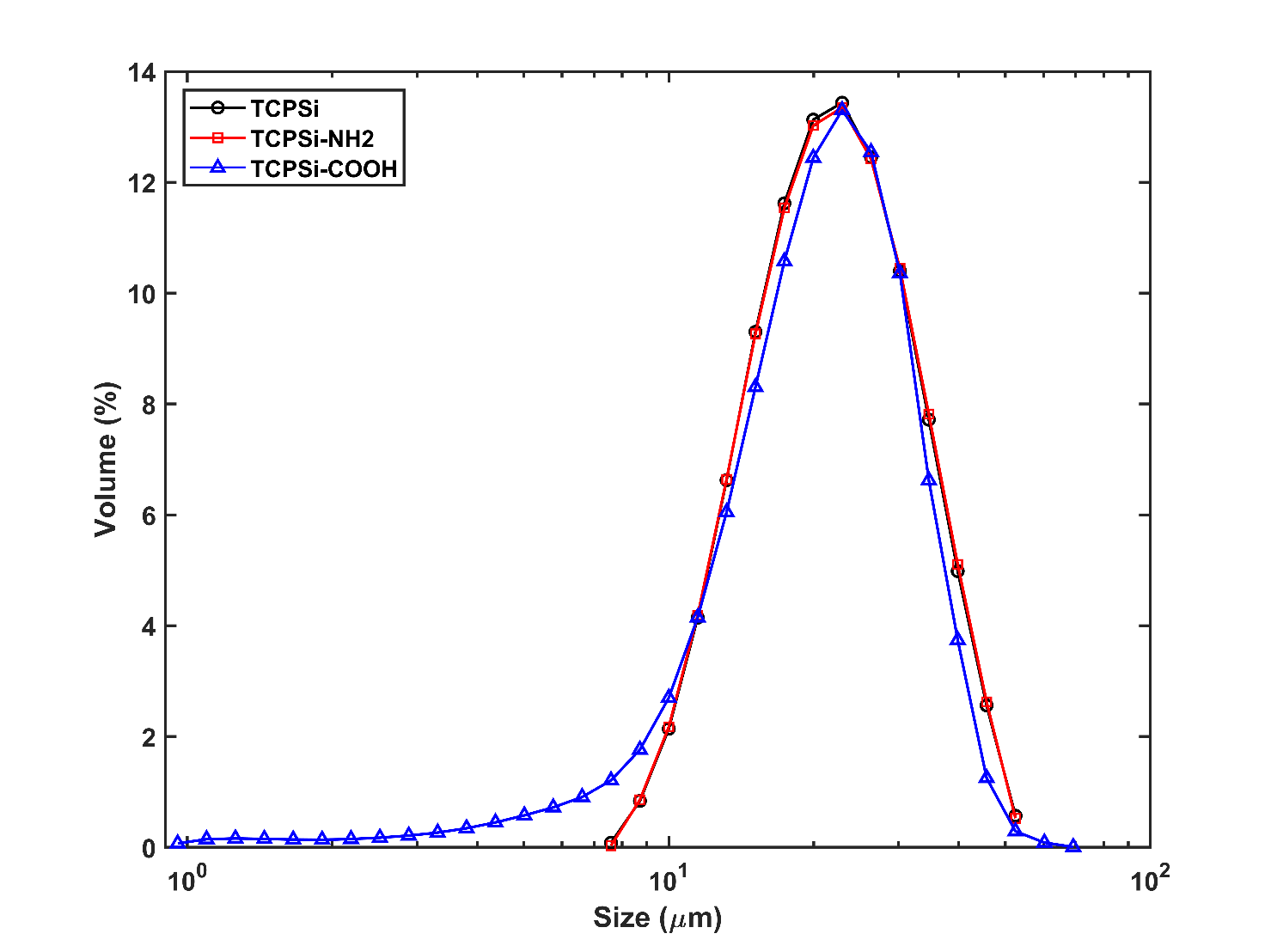


**Fig. S2.** Particle size distribution for TCPSi, TCPSi-NH2 and TCPSi-COOH particles.


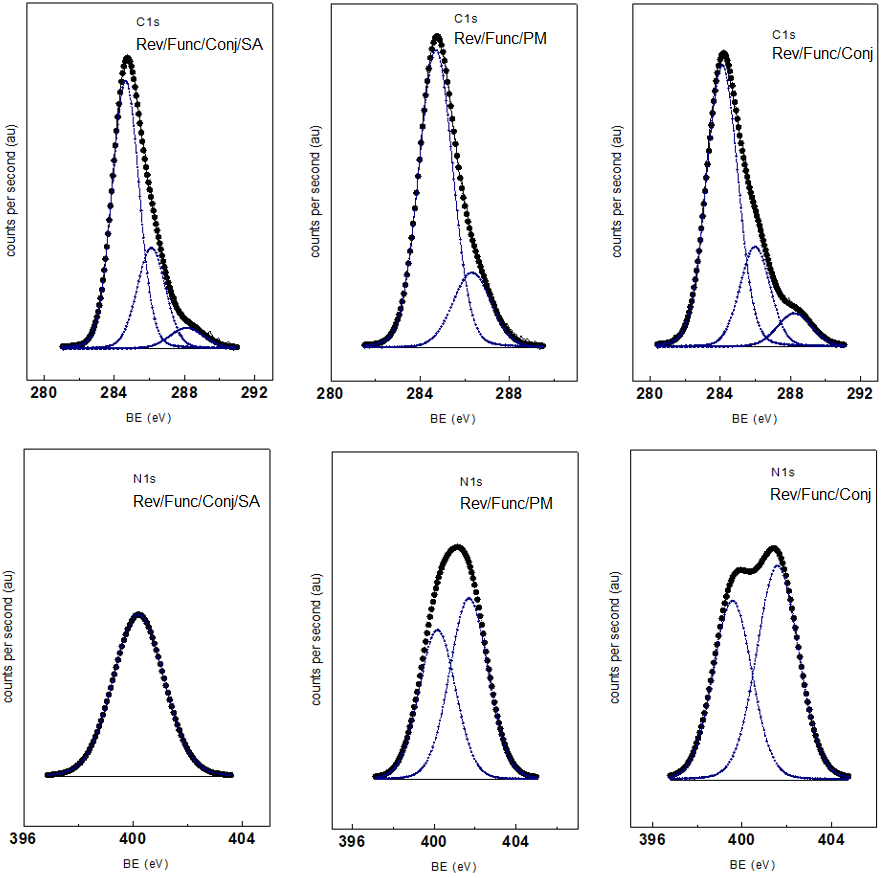


**Fig. S3.** XPS spectra for C1s and N1s with deconvolutions shown.

**Table S1.** Atomic surface composition of TCPSi-CNT samples.

| **Sample** | **C (%at)** | **N (%at)** | **O (%at)** | **Si (%at)** |
| --- | --- | --- | --- | --- |
| **Rev/Func/Conj** | 51.4 | 0.8 | 15.3 | 32.5 |
| **Rev/Func/Conj/SA** | 45.2 | 0.2 | 19.8 | 34.8 |
| **Rev/Func/PM** | 56.3 | 0.3 | 13.0 | 30.4 |


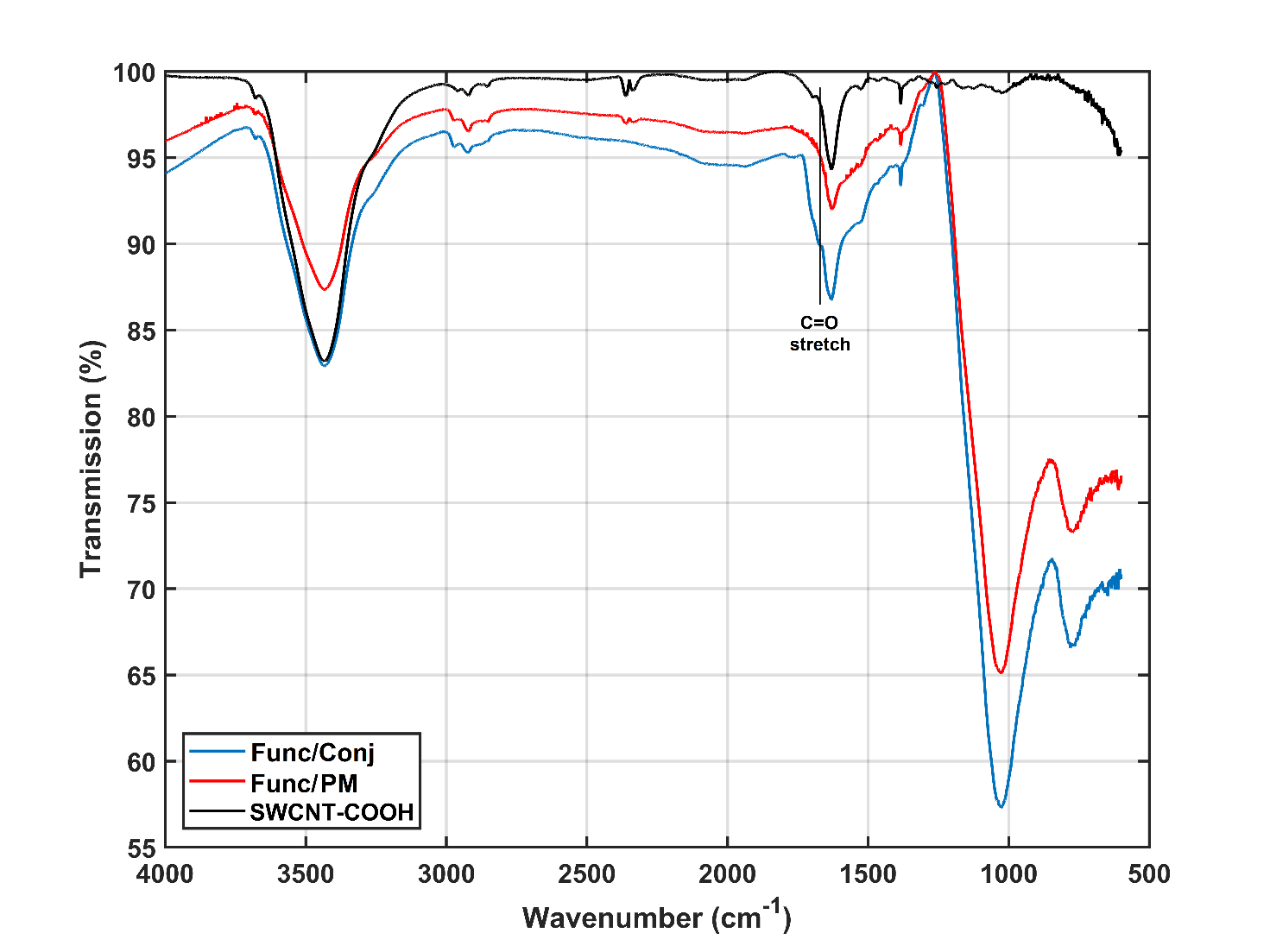


**Fig. S4.** FTIR spectra of Func/Conj, Func/PM and SWCNT-COOH.

**
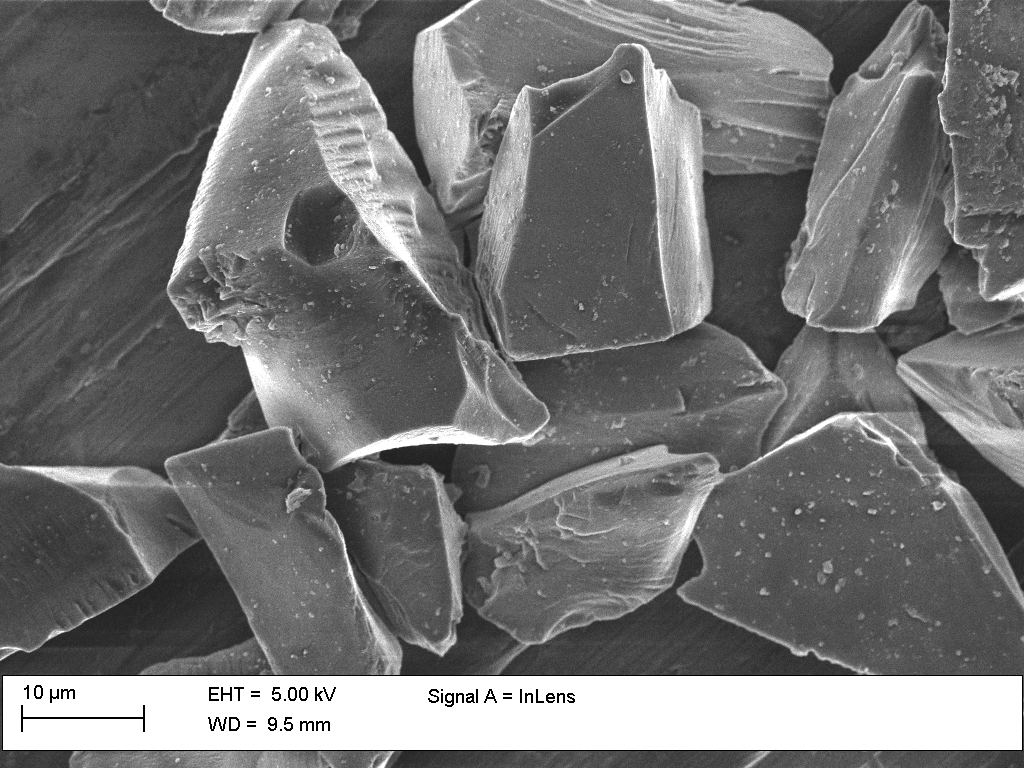
**

**Fig S5.** SEM image of individual PSi particles.


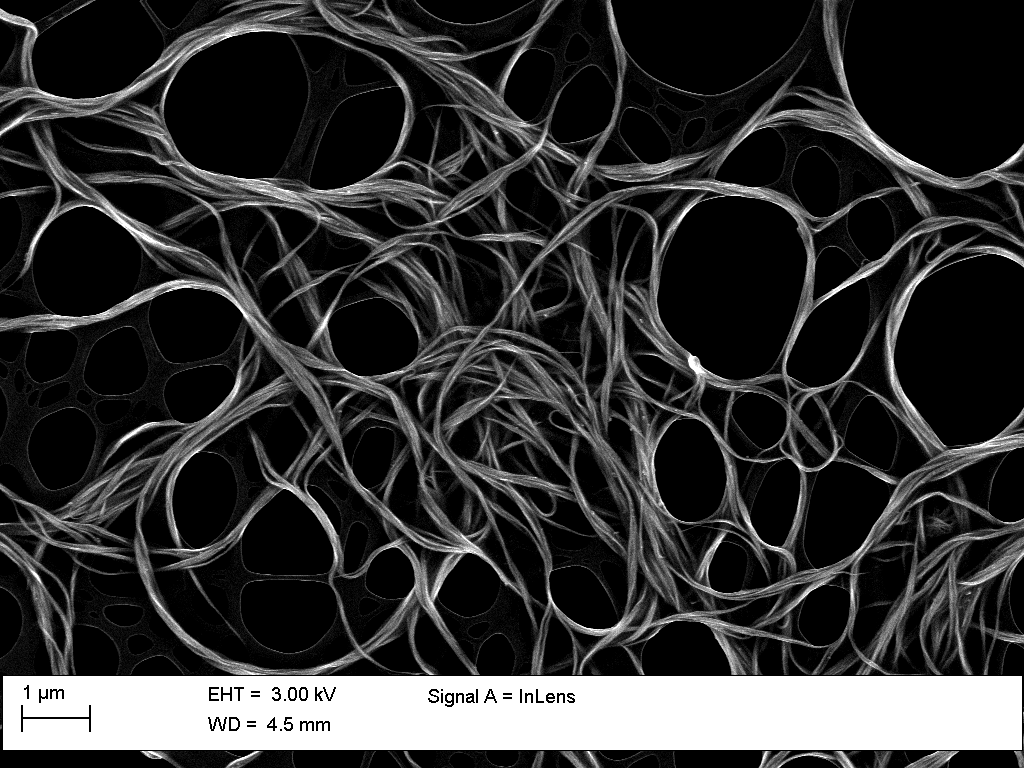


**Fig. S6.** SEM image of CNTs on holey carbon grid.


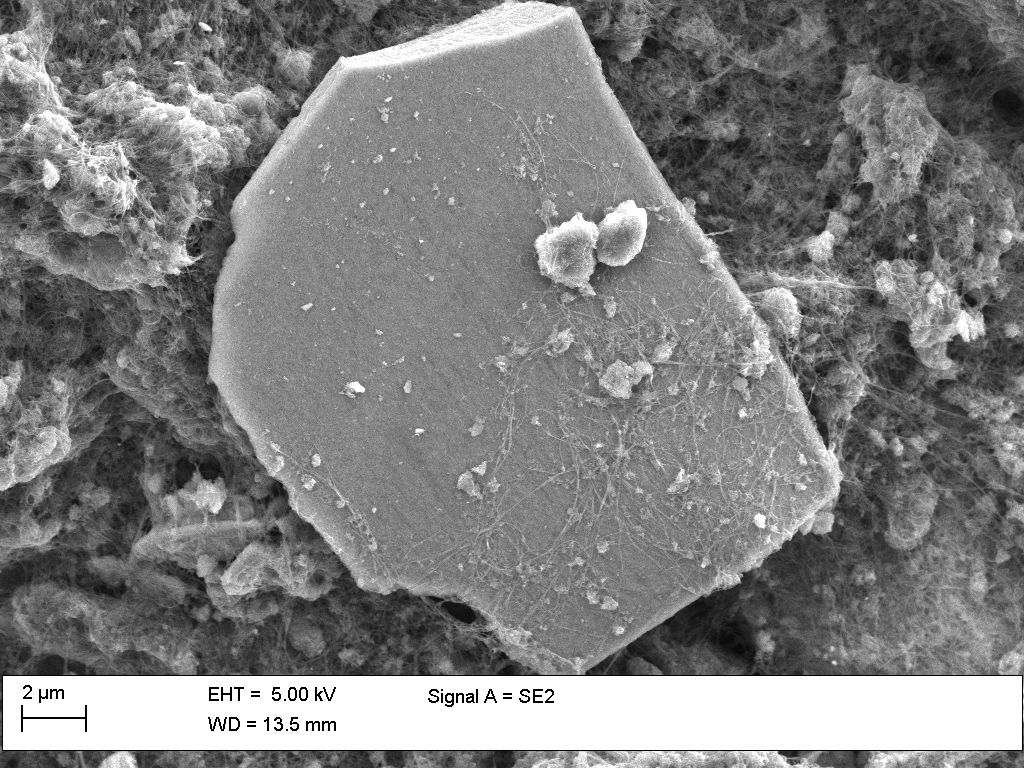


**Fig. S7.** Individual PSi particle partially covered with CNTs.

**
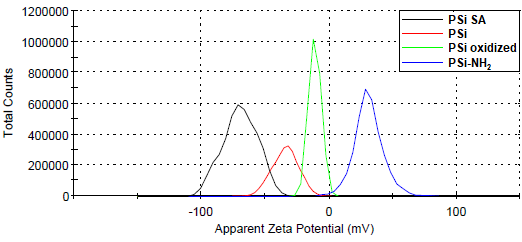
**

**Fig. S8.** Zeta potential distribution of differently functionalized PSi nanoparticles: Porous silicon with SA treatment (PSi SA), as-anodized porous silicon (PSi), chemically oxidized porous silicon (PSi oxidized) and amine-modified porous silicon (PSi-NH_2_).

**
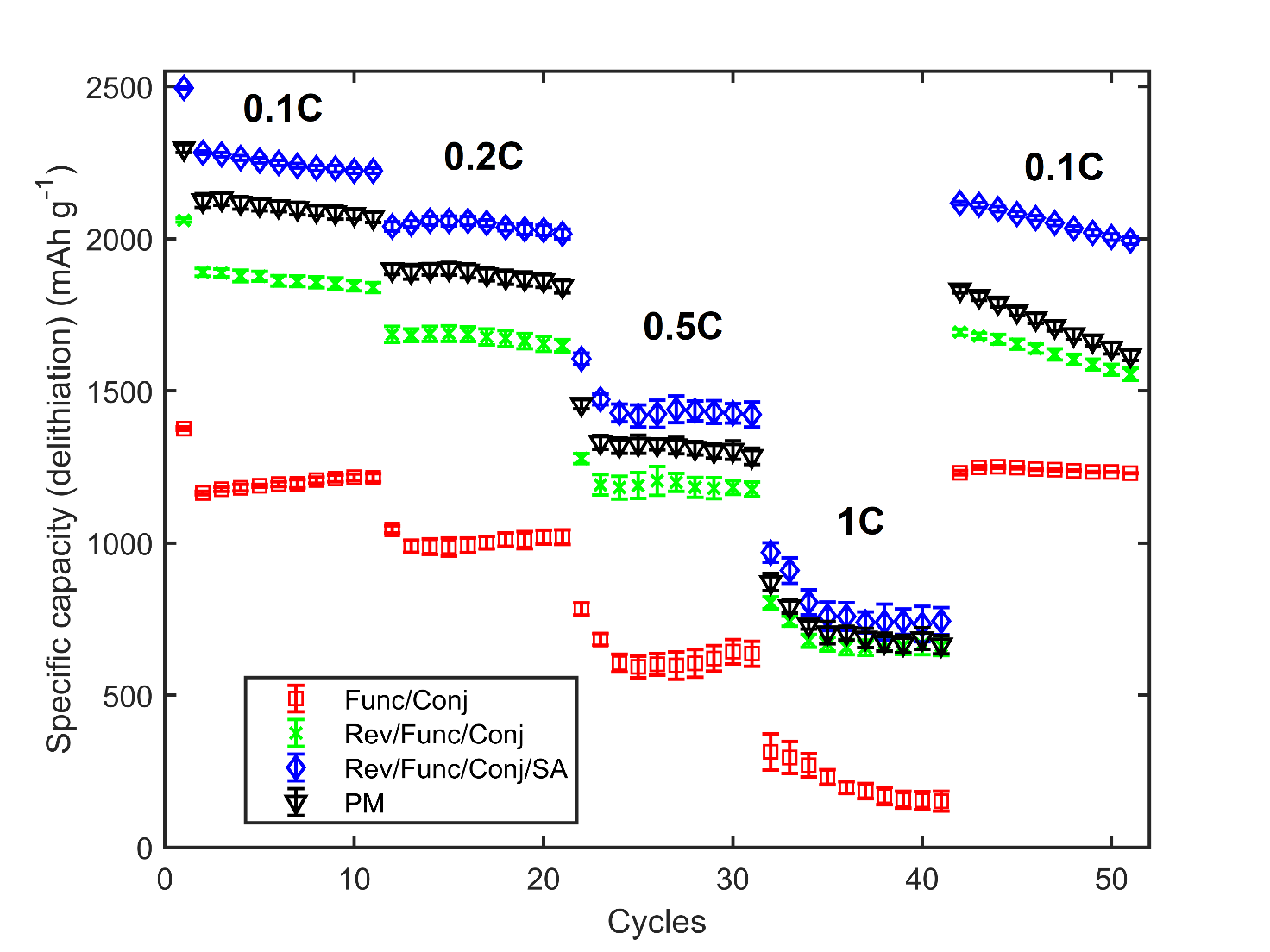
**

**Fig. S9.** Galvanostatic rate capability results for the Func/Conj, Rev/Func/Conj, Rev/Func/Conj/SA and PM samples. The results are shown with average value and standard error (n = 3).


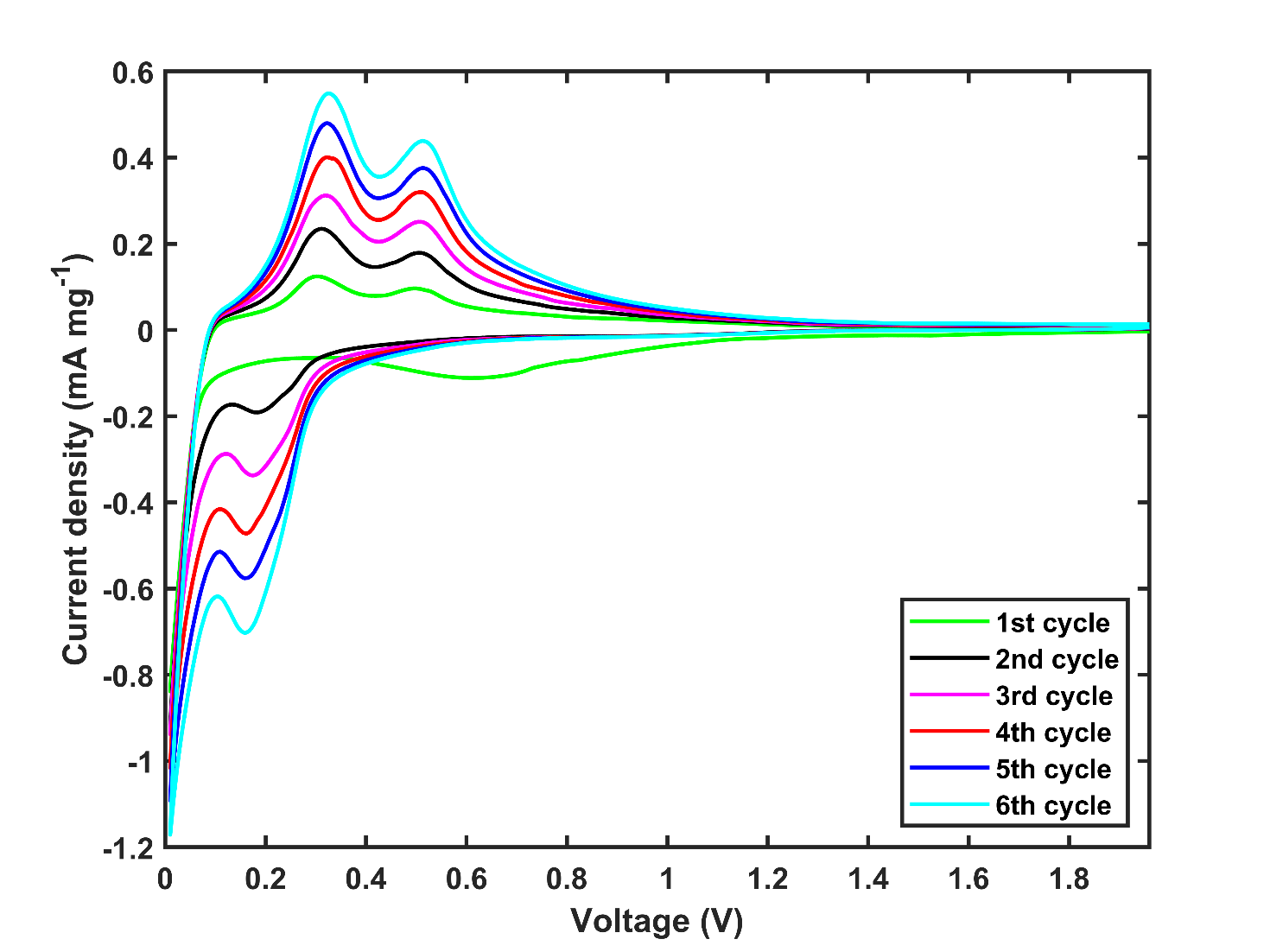


**Fig. S10.** Cyclic voltammetry results for Rev/Func/PM showing the capacity increase during cycling.

**Table S2**. Values for the half-peak potentials calculated from the CV curves.

| **Sample** | **1^st^ cycle**  **Anodic half peak potential 1 (V)** | **1^st^ cycle**  **Anodic half peak potential 2 (V)** | **6th cycle**  **Cathodic half peak potential**  **(V)** | **6^th^ cycle**  **Anodic half peak potential 1 (V)** | **6^th^ cycle**  **Anodic half peak potential 2 (V)** |
| --- | --- | --- | --- | --- | --- |
| PM | 0.252 | 0.273 | 0.227 | 0.272 | 0.289 |
| Func/Conj | 0.239 | 0.232 | 0.255 | 0.250 | 0.236 |
| Rev/Func/Conj | 0.249 | 0.257 | 0.237 | 0.253 | 0.244 |
| Rev/Func/PM | 0.224 | 0.195 | 0.255 | 0.243 | 0.226 |
| Rev/Func/Conj/SA | 0.227 | 0.215 | 0.253 | 0.253 | 0.245 |

**
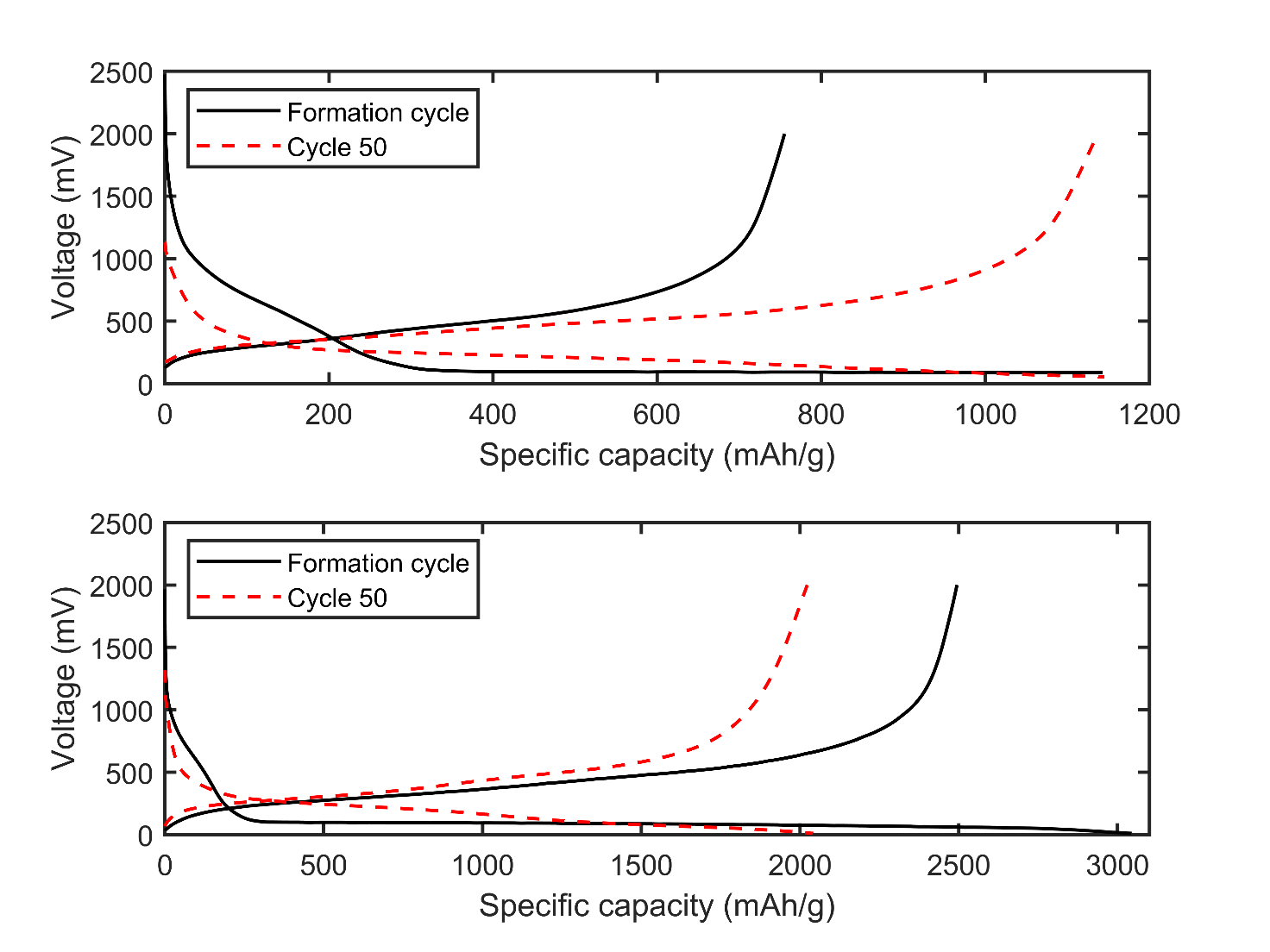
**

**Fig. S11.** Voltage profile for Rev/Func/Conj/SA sample in life cycle testing (above) and rate performance test (below).


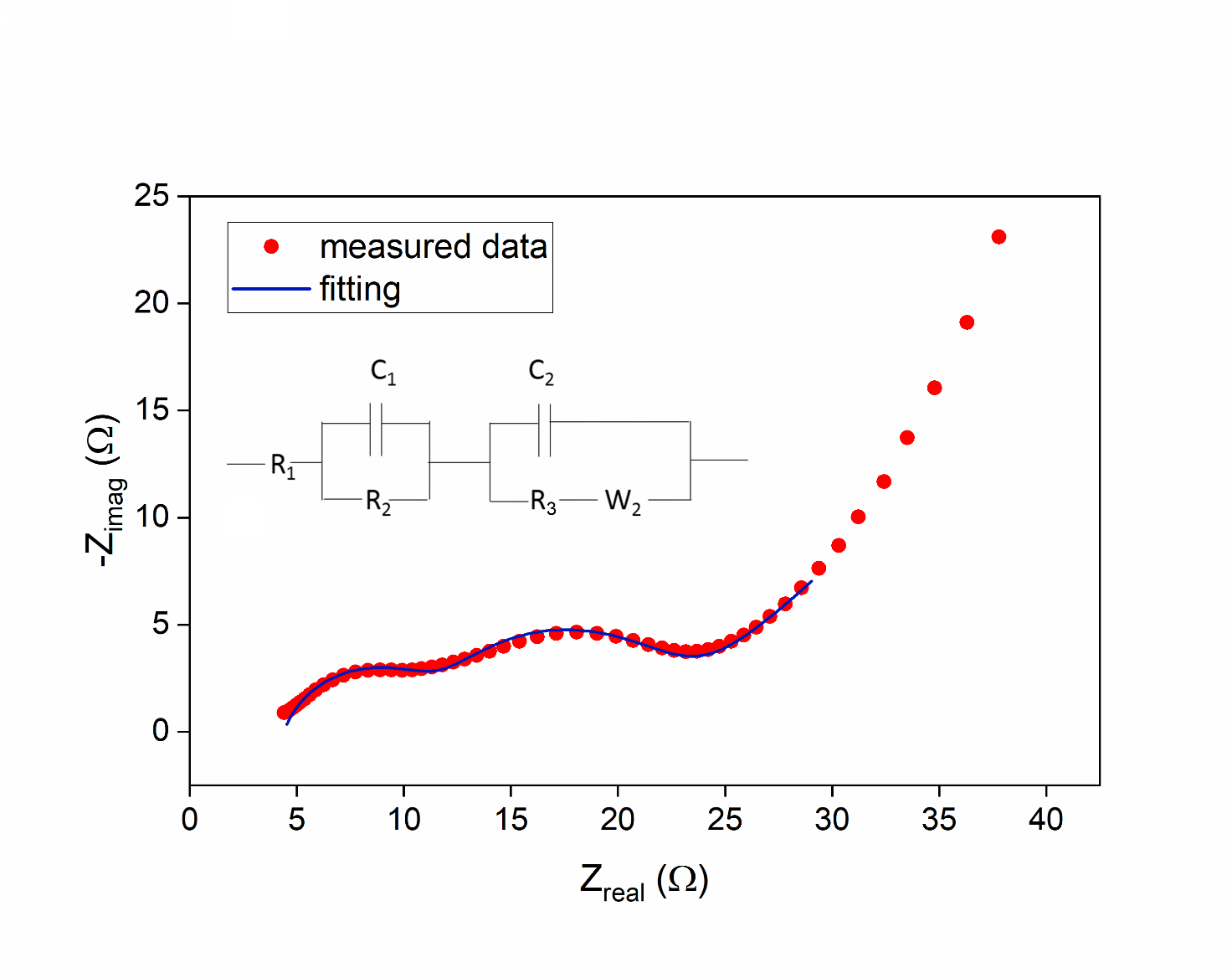


**Fig. S12.** Experimental impedance data with equivalent circuit and fit for Rev/Func/Conj/SA sample after formatting.
